# The Electronic CardioMetabolic Program (eCMP) for Patients With Cardiometabolic Risk: A Randomized Controlled Trial

**DOI:** 10.2196/jmir.5143

**Published:** 2016-05-27

**Authors:** Kristen MJ Azar, Suneil Koliwad, Tak Poon, Lan Xiao, Nan Lv, Robert Griggs, Jun Ma

**Affiliations:** ^1^ Sutter Health Research, Development and Dissemination Walnut Creek, CA United States; ^2^ Palo Alto Medical Foundation Research Institute Palo Alto, CA United States; ^3^ University of California San Francisco San Francisco, CA United States; ^4^ Mills Peninsula Hospital Burlingame, CA United States; ^5^ Stanford University School of Medicine Palo Alto, CA United States; ^6^ University of Illinois Chicago, IL United States

**Keywords:** prevention, lifestyle, metabolic syndrome, cardiovascular disease, behavior change, health technology

## Abstract

**Background:**

Effective lifestyle interventions targeting high-risk adults that are both practical for use in ambulatory care settings and scalable at a population management level are needed.

**Objective:**

Our aim was to examine the potential effectiveness, feasibility, and acceptability of delivering an evidence-based Electronic Cardio-Metabolic Program (eCMP) for improving health-related quality of life, improving health behaviors, and reducing cardiometabolic risk factors in ambulatory care high-risk adults.

**Methods:**

We conducted a randomized, wait-list controlled trial with 74 adults aged ≥18 years recruited from a large multispecialty health care organization. Inclusion criteria were (1) BMI ≥35 kg/m^2^ and prediabetes, previous gestational diabetes and/or metabolic syndrome, or (2) BMI ≥30 kg/m^2^ and type 2 diabetes and/or cardiovascular disease. Participants had a mean age of 59.7 years (SD 11.2), BMI 37.1 kg/m^2^ (SD 5.4) and were 59.5% female, 82.4% white. Participants were randomized to participate in eCMP immediately (n=37) or 3 months later (n=37). eCMP is a 6-month program utilizing video conferencing, online tools, and pre-recorded didactic videos to deliver evidence-based curricula. Blinded outcome assessments were conducted at 3 and 6 months postbaseline. Data were collected and analyzed between 2014 and 2015. The primary outcome was health-related quality of life. Secondary outcomes included biometric cardiometabolic risk factors (eg, body weight), self-reported diet and physical activity, mental health status, retention, session attendance, and participant satisfaction.

**Results:**

Change in quality of life was not significant in both immediate and delayed participants. Both groups significantly lost weight and reduced waist circumference at 6 months, with some cardiometabolic factors trending accordingly. Significant reduction in self-reported anxiety and perceived stress was seen in the immediate intervention group at 6 months. Retention rate was 93% at 3 months and 86% at 6 months post-baseline. Overall eCMP attendance was high with 59.5-83.8% of immediate and delayed intervention participants attending 50% of the virtual stress management and behavioral lifestyle sessions and 37.8-62.2% attending at least 4 out of 7 in-person physical activity sessions. The intervention received high ratings for satisfaction.

**Conclusions:**

The technology-assisted eCMP is a feasible and well-accepted intervention and may significantly decrease cardiometabolic risk among high-risk individuals.

**Trial Registration:**

Clinicaltrials.gov NCT02246400; https://clinicaltrials.gov/ct2/show/NCT02246400 (Archived by WebCite at http://www.webcitation.org/6h6mWWokP)

## Introduction

The risk for cardiometabolic diseases remains high among US adults. Although the rates of obesity, a major contributor to this risk, are leveling, up to 35% of the population continue to be classified as obese (body mass index [BMI] ≥30 kg/m^2^) [1]. Addressing the prevention and treatment needs of the population remains a challenge. Effective and scalable health care systems are urgently needed to promote patient-centered population health management among patients who either have or are at risk for cardiometabolic diseases.

Lifestyle intervention integrated into ambulatory care has consistently been emphasized as a crucial approach to cardiometabolic risk reduction. In the context of obesity, lifestyle modification has been shown to produce significant risk reduction even with modest weight loss (3-5%) [2]. While intensive lifestyle interventions to reduce cardiometabolic risk have been shown to be beneficial, much work is needed to translate efficacious interventions into practical and sustainable programs that can be offered by the existing health care infrastructure. Patient-centered population health management to prevent and control cardiometabolic disease requires scalable and sustainable lifestyle interventions.

Technology-assisted approaches that are based in ambulatory care may increase the potential for widespread reach and adoption, resulting in improved long-term effectiveness and a shift towards a population based management model. Growing evidence [3-6] suggests that technology-assisted clinical tools and approaches can both increase access and decrease cost for clinic-based disease prevention and management programs that traditionally place a large burden on personnel and resources. While evidence suggests that technology-assisted lifestyle interventions for weight loss are effective, best practices remain unknown [7]. Shortcomings of existing interventions include low level of pragmatic methodology and use of technology that is not publicly available [7]. Further, there is growing concern that increased emphasis on automated online delivery modalities for lifestyle intervention can potentially fail because they sacrifice important face-to-face interactions between health care providers and patients, and among patients in group settings [8]. With this in mind, efforts to test the utility of technology-based interventions specifically for ambulatory care are underway, using widely available and low-cost tools to improve delivery mechanisms, enhance patient-provider communication, and preserve virtual face-to-face interactions while optimizing access and reach.

The purpose of this study was to examine the potential effectiveness, feasibility, and acceptability of an evidence-based group lifestyle intervention via real-time videoconferencing with other technology-assisted tools to reduce obesity and cardiometabolic risk factors among high-risk individuals in an ambulatory care setting. We hypothesized that participants would report improvements in health-related quality of life at 3 months post-baseline, compared to the delayed control group. For secondary outcomes, we hypothesized that cardiometabolic risk reduction would result in the immediate intervention group at 3 months and that these findings would be replicated in the delayed group at 6 months, while the immediate group would show continued improvement.

## Methods

### Study Design

The Electronic CardioMetabolic Program (eCMP) pilot study was a randomized, wait-list controlled trial among patients who either had or were at high risk for developing type 2 diabetes and/or cardiovascular disease (cardiometabolic disease). The primary end point was originally intended to be 6 months post baseline. The original intent had been for the wait-list control group to not begin the intervention until the immediate group completed the 6-month intervention, allowing for between-group comparisons at 3 and 6 months. However, this was a pilot study and logistic challenges including limited duration and funding were encountered after the study began. In order to comply with logistical limitations, we phased in the wait-list control sooner and therefore were able to compare outcomes between groups (intervention vs control) at 3 months post baseline and compare changes in outcomes within the immediate intervention group at 6 months post baseline and for the delayed-intervention group at 3 months post intervention (the mid-way point). We looked at reproducibility of early/mid-intervention effects between the first 3 months of participation and saw similar patterns of change as well as a continuation of trends.

### Recruitment and Participants

Participants were recruited from an outpatient multispecialty group practice organization in Northern California. High-risk adults (≥18 years old) in need of primary prevention of cardiovascular disease and/or diabetes (body mass index [BMI] ≥35 kg/m^2^ and pre-diabetes, previous gestational diabetes and/or metabolic syndrome) or secondary prevention (BMI ≥30 kg/m^2^ and type 2 diabetes and/or cardiovascular disease) were invited to participate. Participants were required to be proficient in written and spoken English and to have access to the Internet to allow for remote self-tracking, viewing of online materials and resources (eg, pre-recorded didactic videos) as well as participation in videoconference group visits. Exclusion criteria included type 1 diabetes or insulin dependence, pregnancy or active breastfeeding at the time of enrollment, current treatment for a serious medical condition (ie, cancer, except non-melanoma skin cancer), presence of any safety concerns related to significant physical or mental health issues, or life expectancy less than 12 months.

Potential eligible participants were identified through electronic health records. Participants were first screened for eligibility via phone or online and were then invited to attend the baseline assessment visit where informed consent was obtained. During the baseline visit, participants were given more information regarding the study and baseline clinical measures were obtained and eligibility was confirmed prior to randomization. Participants were interviewed at each follow-up visit about possible adverse events during the past 3 months, and the study physician adjudicated the events per study safety protocol. Data were collected and analyzed between June 2014 and January 2015 in Burlingame, California.

**Figure 1:**
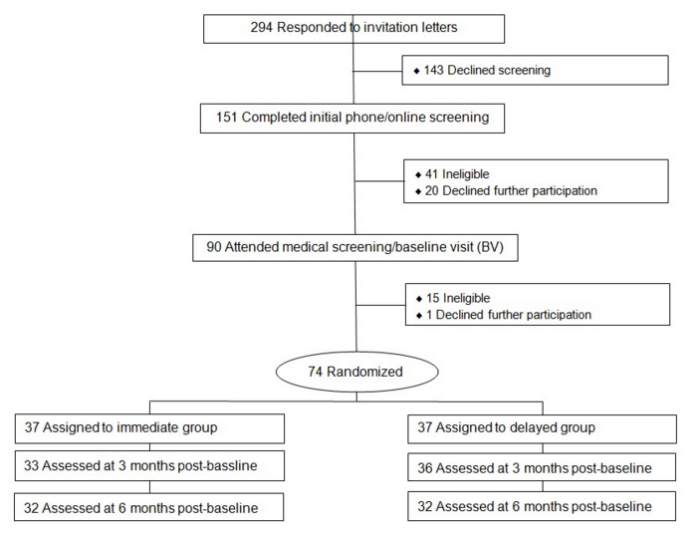
CONSORT diagram.

The study was approved by the Palo Alto Medical Foundation Institutional Review Board. Of the 294 patients who responded to recruitment letters after their primary care provider approved study contact, 164 patients declined participation and 56 were ineligible. This process yielded the target enrollment of 74 eligible and consenting participants (see Figure 1).

### Randomization and Allocation Concealment

We applied our published dynamic block randomization method [9] to assure better than chance between-treatment balance across 5 prognostic factors (age, sex, race/ethnicity, BMI, and primary prevention). The method automatically ensures allocation concealment. Participants were randomized to receive the 6-month eCMP lifestyle intervention either immediately upon randomization (n=37) or after a 3-month wait period (n=37). A designated study staff person who did not have the ability to influence the allocation system’s execution performed randomization. While study group assignment was identifiable to participants and interventionists, blinding was otherwise maintained for data collection, outcome adjudication, and data analysis.

### Description of the eCMP Intervention

The goal of the eCMP is to reduce cardiometabolic risk by improving diet, physical activity, and mental health behaviors through lifestyle change. eCMP is a 6-month comprehensive program relying on weekly face-to-face group meetings via video conferencing and the delivery of evidence-based curricula using online tools and pre-recorded didactic videos presented by physicians, nutritionists, exercise physiologists, and lifestyle coaches. Technology-assisted features include (1) portable and/or wearable mobile devices for data collection (eg, Fitbit and Withings Smart Scale WS-30 [wireless scale]), (2) a comprehensive online platform and participant portal for hosting program materials (eg, homework assignments, didactic videos, and calendars), and (3) virtual small groups via real-time, encrypted, Web-based videoconferencing (see Table 1). Wireless scales were provided to the immediate intervention groups only. Technology-assisted tools for self-tracking and participation were provided for use during the study period. All participants attended an in-person orientation session post baseline assessment and prior to their first group visit. At the orientation, they participated in a technology-training workshop and received all intervention tools and materials. There were a total of 24 virtual group sessions offered alternatively between the stress management and behavioral lifestyle component and 7 in-person sessions for group-based physical activity.

**Table 1:** eCMP curriculum contents and delivery modalities in ambulatory care settings.

Component	Function	Features
Evidence- and theory-based curriculum^a^	Lifestyle modification and behavior change content	Weight management
		Healthy eating
		Physical activity
		Stress management
Online platform and participant portal	Hosting program materials and participant-coach communication	Didactic videos
		Homework assignments
		Calendar/schedule
		Other educational resources
Mobile monitoring devices	Participant self-monitoring, bio-feedback, and remote data capture	Wireless body scale^b^
		Pedometer
Coach-led virtual small group sessions	Curriculum content delivery for weight management, healthy eating, and stress management	Weekly (24) sessions
		Real-time, encrypted, Web-based video conferencing
Coach-led in-person sessions	Exercise curriculum content delivery	Periodic (7) sessions

^a^Curriculum used includes Diabetes Prevention Program Group Lifestyle Balance (weight management and healthy eating), Active Living Every Day (physical activity), and Heart Matters (stress management).

^b^Immediate intervention group only.

### Stress Management Component

Interactive sessions for stress management consisted of biweekly virtual small group meetings of 7-10 participants. Each 1-hour session was facilitated by a trained health coach and was based on a proprietary, evidence-based [10-17] stress management curriculum called *Heart Matters*. Self-awareness is the foundation of the *Heart Matters* program, which includes these topics: belief systems, emotional responses to experiences, anger, hostility, time pressure, mindfulness, self-esteem, and forgiveness. Prior to each stress management session, participants were instructed to view an accompanying, supplemental video available via the online platform.

### Behavioral Lifestyle Component

Interactive sessions for diet counseling consisted of biweekly virtual small group meetings of 7-10 participants. A registered dietitian facilitated each 1-hour session, using the core curriculum of the evidence-based Group Lifestyle Balance program developed by researchers at the University Of Pittsburgh [18,19]. Participants were asked to track their daily dietary intake via an Internet/mobile-based self-monitoring app.

### Physical Activity Component

Participants were provided with instructional videos available through the online platform that encourage participants to identify opportunities throughout their day for increasing physical activity. The video content and exercise modalities were adapted from the Active Living Every Day program designed to increase physical activity among sedentary individuals [20,21]. In addition to the videos, participants were encouraged to attend 7 drop-in exercise sessions and to track their daily steps with a Fitbit pedometer.

### Outcome Measures

All outcome assessors were trained to perform the measurements and interviews per standardized protocols and procedures at baseline and at 3 and 6 months post baseline. The primary outcome, overall health-related quality of life, was measured by the Short Form-8 Health Survey (SF-8), an 8-item version of the SF-36 [22]. Secondary outcomes included biometric cardiometabolic risk factors (eg, body weight), self-reported diet and physical activity, and mental health status, retention, session attendance, and participant satisfaction. Published protocols were used to obtain height (baseline only), weight, waist circumference, and blood pressure measurements [23,24]. Participants also completed fasting blood draws at the onsite clinical laboratory for assays of plasma lipid profile. Dietary intake was assessed using multiple-pass 24-hour diet recall [25] of one typical day over the phone with participants on the Windows-based Nutrition Data System for Research (NDSR; Nutrition Coordinating Center, University of Minnesota). Diet quality was assessed using a composite Dietary Approaches to Stop Hypertension (DASH) concordance index (range 0-9) calculated by summing 9 nutrients, including total fat, saturated fat, protein, cholesterol, fiber, magnesium, calcium, sodium, and potassium [26]. Physical activity was assessed using the Stanford 7-Day Physical Activity Recall interview [27]. Stress and mental health measures included the Patient Health Questionnaire-9 (PHQ-9), Generalized Anxiety Disorder Scale (GAD-7), and Perceived Stress Scale. The PHQ-9 is a 9-item depression symptom assessment with scores ranging from 0-27. PHQ-9 scores of 5-9, 10-14, 15-19, and 20-27 represent mild, moderate, moderately severe, and severe depression, respectively [28,29]. The GAD-7 is a valid and reliable 7-question scale for screening generalized anxiety disorder and strongly associated with multiple domains of functional impairment [30]. The Perceived Stress Scale, a 14-item instrument, is a global measure of perceived stress that asks participants to report how often their lives seem to be uncontrollable or overloaded during the last year [31].

Attendance was tracked for all participants for the entire 6-month study period. Anonymous feedback and satisfaction ratings with the eCMP intervention components were obtained from participants online through survey questions ranked on a 5-point Likert scale with 1 indicating “not at all satisfied” and 5 indicating “extremely satisfied.” The survey included questions pertaining to session-related components (ie, technology, coach, and group dynamics) and home activities and materials (eg, self-monitoring activities, video/DVD, handouts, and the “virtual package”). All eCMP participants (both arms) were encouraged by the coach to complete the survey after each virtual group and/or in-person session (31 sessions total).

### Statistical Analysis

Frequencies and percentages were calculated for describing categorical variables, and means and standard deviations were used for continuous variables. Intention-to-treat analyses of between-group and within-group differences in primary and secondary outcomes were tested in a repeated-measures mixed-effects linear model with adjustment of the baseline value of the outcome of interest. Between-group differences (immediate intervention vs delayed intervention) were examined at 3 months post baseline. Within-group differences for both immediate intervention and the delayed-intervention group were assessed at 6 months post baseline. All analyses were conducted using SAS, version 9.3.

## Results

### Retention and Baseline Characteristics

Patients were recruited from March 21, 2014, through May 13, 2014. Follow-up was completed on January 2, 2015. Of the 74 randomized participants, 89% (33/37) of intervention and 97% (36/37) of delayed intervention participants were assessed at 3 months and 86% (32/37) and 86% (32/37) at 6 months (Figure 1). There was no discernable pattern to the attrition. Participants were 59.7 (11.2) years old, mostly female, non-Hispanic white, and severely obese (see Table 2). At baseline, 27% (20/74) of participants were enrolled for primary prevention and 73% (54/74) for secondary prevention.

**Table 2:** Baseline characteristics.

		All (N=74)	Immediate (n=37)	Delayed (n=37)
Age in years, mean (SD)		59.7 (11.2)	59.6 (11.9)	59.8 (10.5)
Body mass index, kg/m^2^, mean (SD)		37.1 (5.4)	37.0 (5.7)	37.3 (5.2)
Female, %		59.5	59.5	59.5
**Race, %**				
	Hispanic	5.4	5.4	5.4
	Non-Hispanic white	82.4	83.8	81.1
	Non-Hispanic black	4.1	0	8.1
	Asian/Pacific Islander	2.7	2.7	2.7
	Other	5.4	8.1	2.7
Primary prevention^a^, %		27.0	27.0	27.0
Secondary prevention^b^, %		73.0	73.0	73.0

^a^Primary prevention is defined as BMI ≥35 kg/m^2^and prediabetes and/or metabolic syndrome.

^b^Secondary prevention is defined as BMI ≥30 kg/m^2^ and type 2 diabetes and/or cardiovascular disease.

### Quality of Life, Weight, and Cardiometabolic Risk Factors

Change in health-related quality of life was not significant in both immediate and delayed participants. The mean weight loss was -2.3 kg (-2.0% of baseline) in immediate participants versus -0.2 kg (-0.01%) in delayed controls at 3 months. The between-group mean difference in change was -2.1 kg (95% CI -4.3 to 0.1) (see Table 3). At 6 months post baseline, the immediate group achieved significant weight loss of -3.1 kg (95% CI -4.7 to -1.5), achieving a 2.8% weight reduction. Equivalently, immediate intervention participants had greater reductions in BMI than delayed controls at 3 months and the net reduction was -0.7 kg/m^2^ (95% CI -1.5 to -0.02). Delayed controls achieved comparable BMI reductions at 6 months. At 6 months, the immediate intervention group achieved an overall mean reduction of -1.0 kg/m^2^ (95% CI -1.5 to -0.5).

Differences in waist circumference were not significant between the immediate and delayed groups at 3 months, with a decrease of -0.6 cm (95% CI -3.8 to 2.6). The immediate intervention group showed a significant reduction in waist circumference at 6 months post baseline. Participants had normal mean blood pressure, and fasting plasma lipids at baseline, with non-significant but consistent tendency of improvements in the immediate relative to the delayed group at 3 months and inconsistent changes due to insufficient sample size (n=19) for the immediate group at 6 months (see Tables 3 and 4).

**Table 3:** Changes in quality of life, anthropometric, blood pressure, diet, physical activity, and stress and mental health at 3 and 6 months.

		Baseline, mean (SD)	Change from baseline to 3 months, mean (95% CI)			Change from 3 to 6 months, mean (95% CI)		Change from baseline to 6 months, mean (95% CI)
		All (N=74)	Immediate (intensive phase) (n=33)	Delayed (no intervention) (n=36)	Between-group difference, difference in change	Immediate (maintenance phase) (n=32)	Delayed (intensive phase) (n=32)	Immediate (intensive+ maintenance) (n=32)
**Quality of life**								
	SF-8 mental component	49.2 (8.1)	-1.3 (-4.0 to 1.5)	1.5 (-1.1 to 4.1)	-2.8 (-6.6 to 1.0)	3.0 (-0.7 to 6.6)	1.3 (-2.3 to 4.8)	1.7 (-1.3 to 4.6)
	SF-8 physical component	45.1 (7.2)	2.3 (-0.4 to 5.1)	0.9 (-1.7 to 3.6)	1.4 (-2.4 to 5.2)	-2.6 (-5.8 to 0.6)	1.7 (-1.4 to 4.8)	-0.2 (-3.1 to 2.7)
**Anthropometric and blood pressure**								
	BMI, kg/m^2^	37.1 (5.4)	-0.7 (-1.3 to -0.2)	0 (-0.5 to 0.5)	-0.7 (-1.5 to -0.02)^a^	-0.3 (-0.7 to 0.2)	-0.6 (-1.0 to -0.1)^b^	-1.0 (-1.5 to -0.5)^b^
	Weight, kg	107.4 (18.8)	-2.3 (-3.9 to -0.7)	-0.2 (-1.7 to 1.3)	-2.1 (-4.3 to 0.1)	-0.8 (-2.1 to 0.4)	-1.6 (-2.9 to -0.4)^b^	-3.1 (-4.7 to -1.5)^b^
	Weight change, %	0	-2.0 (-3.5 to -0.6)	0 (-1.4 to 1.4)	-2.0 (-4.0 to -0.1)^a^	-0.8 (-1.9 to 0.4)	-1.5 (-2.6 to -0.4)^b^	-2.8 (-4.2 to -1.4)^b^
	Waist circumference, cm	119.6 (13.9)	-1.9 (-4.2 to 0.4)	-1.3 (-3.5 to 0.9)	-0.6 (-3.8 to 2.6)	-2.2 (-4.2 to -0.2)^b^	-1.1 (-3.0 to 0.8)	-4.1 (-6.5 to -1.7)^b^
	Systolic blood pressure	124.0 (10.1)	0.7 (-2.2 to 3.6)	1.1 (-1.7 to 3.9)	-0.4 (-4.4 to 3.6)	-0.3 (-3.8 to 3.1)	-2.7 (-6.0 to 0.6)	0.3 (-2.7 to 3.4)
	Diastolic blood pressure	72.6 (10.7)	2.9 (0.6-5.3)	2.6 (0.4-4.8)	0.3 (-2.9 to 3.6)	2.0 (-1.0 to 5.0)	2.8 (-0.5 to 6.1)	5.0 (2.5 to 7.4)^b^
**Diet and physical activity **								
	DASH score^c^	2.4 (1.4)	0.4 (-0.1 to 0.8)	0.1 (-0.4 to 0.6)	0.3 (-0.4 to 0.9)	-0.3 (-0.9 to 0.4)	0.3 (-0.4 to 0.9)	0.1 (-0.4 to 0.6)
	Stanford 7-day Physical Activity Recall (metabolic equivalents)	680.5 (634.5)	652.8 (236.8-1068.7)	103.6 (-294.8 to 501.9)	549.2 (-26.8 to 1125.2)	-708.5 (-1224.8 to -192.2)^b^	-419.2 (-926.1 to 87.7)	-55.7 (-477.7 to 366.3)
**Stress management**								
	PHQ-9	5.2 (4.1)	-1.1 (-2.2 to 0)	-0.8 (-1.9 to 0.2)	-0.3 (-1.8 to 1.2)	0.3 (-0.9 to 1.6)	-0.8 (-1.9 to 0.4)	-0.8 (-1.9 to 0.4)
	GAD-7	3.4 (3.1)	0.2 (-0.7 to 1.2)	-0.5 (-1.5 to 0.4)	0.8 (-0.6 to 2.1)	-1.2 (-2.4 to 0)	0.4 (-0.8 to 1.5)	-1.0 (-2.0 to 0)
	Perceived Stress Scale	13.1 (6.1)	0.3 (-1.3 to 2.0)	-1.2 (-2.7 to 0.4)	1.5 (-0.8 to 3.8)	-1.7 (-3.9 to 0.5)	0.4 (-1.7 to 2.6)	-1.4 (-3.2 to 0.4)

^a^ *P*<.05 between group difference.

^b^ *P*<.05 within group difference.

^c^DASH scores were calculated based on combining nine nutrient targets (ie, total fat, saturated fat, protein, cholesterol, fiber, magnesium, calcium, sodium, and potassium). The intermediate target of each nutrient was halfway between the DASH target and population mean (based on the National Health and Nutrition Examination Surveys 2007-2008, latest data available at the inception of this study). For a nutrient, participants reaching the DASH target were assigned one point, those reaching the intermediate target were assigned a half-point, and those not meeting the intermediate target were given 0 point. The DASH score was the sum of points for all 9 nutrients.

**Table 4:** The changes in fasting plasma lipids by groups at 3 and 6 months.

	Baseline, mean (SD)	Change from baseline to 3 months, mean (95% CI)			Change from 3 to 6 months, mean (95% CI)		Change from baseline to 6 months, mean (95% CI)
	All (N=63)	Immediate (intensive phase) (n=22)	Delayed (no intervention) (n=32)	Between-group difference, difference in change	Immediate (maintenance phase) (n=13)	Delayed (intensive phase) (n=24)	Immediate (intensive+ maintenance) (n=19)
TC	175.1 (40.6)	-5.0 (-19.3 to 9.2)	5.4 (-5.8 to 16.7)	-10.5 (-28.7 to 7.7)	16.1 (3.1-29.1)	-0.8 (-10.4 to 8.7)	11.1 (-2.2 to 24.3)
HDL-C	49.7 (16.3)	5.0 (1.8-8.2)	2.5 (0.1- 4.9)	2.5 (-1.5 to 6.5)	2.1 (-1.3 to 5.5)	0.4 (-2.2 to 3.0)	7.2 (4.3-10.1)^a^
LDL-C	98.5 (36)	-10.7 (-23 to 1.5)	0.6 (-9.0 to 10.2)	-11.3 (-26.9 to 4.3)	16.0 (4.2-27.9)	1.8 (-7.0 to 10.6)	5.3 (-6.1 to 16.7)
TC:HDL ratio	3.8 (1.1)	-0.4 (-0.8 to 0.1)	-0.1 (-0.4 to 0.3)	-0.3 (-0.8 to 0.3)	0.2 (-0.1 to 0.4)	0 (-0.2 to 0.2)	-0.2 (-0.6 to 0.2)
Tri-glyceride	134.5 (61.5)	-0.5 (-23.8 to 22.8)	-7.1 (-28.9 to 14.6)	-12.6 (-42.5 to 17.3)	-6.7 (-28.1 to 14.8)	-14.8 (-30.5 to 0.9)	-7.1 (-28.9 to 14.6)

^a^ *P*<.05 within-group difference.

### Diet and Physical Activity Behaviors

DASH score and assessment of leisure-time physical activity of at least moderate intensity did not show statistically significant improvement for either group at 3 and 6 months (see Table 3).

### Stress and Mental Health Measures

None of the stress and mental health measures (ie, PHQ-9, GAD-7, and perceived stress scale) had significant improvement in the immediate relative to the delayed group at 3 months (see Table 3). The mean changes in PHQ-9, GAD-7, and perceived stress scale showed non-significant but consistent tendency of improvements at 6 months for the immediate intervention group.

### Intervention Attendance

Attendance at virtual and in-person group sessions varied by component. For the stress management component, attendance was higher, with 65% (24/37) attendance and 78% (29/37) attendance of immediate and delayed intervention participants, respectively, attending at least half of the 12 total sessions offered. For the behavioral lifestyle component, a majority (60%, 22/37) of immediate intervention participants attended at least half of the sessions offered and 22% (8/37) attended at least 80% of the 12 total sessions offered over the 6-month intervention. Similarly, a large majority (84%, 31/37) of delayed intervention participants attended at least half of the sessions offered, and nearly half (43%, 16/37) of them attended at least 80% of sessions offered. Further, 19% (7/37) of immediate intervention participants compared to 49% (18/37) of delayed intervention participants attended at least 80% of total sessions for stress management offered. For the physical activity component, 38% (14/37) and 62% (23/37) of immediate and delayed intervention participants, respectively, attended at least 4 of the 7 offered sessions. Adherence to 80% of sessions offered was 16% (6/37) for immediate intervention and 11% (4/37) for delayed intervention group.

### Participant Satisfaction and Feedback

Among all participants (both arms) who attended sessions, 39.10% (461/1179) completed the participant satisfaction surveys. Results between the immediate and delayed intervention group were similar (see Table 5). Overall satisfaction was high with scores ranging from mean 4.1 (SD 0.9) to 4.4 (SD 0.7), with health coaches and facilitators rated as the highest satisfying component compared to other components. More than half of participants indicated that they were satisfied (42.4%, 189/446) or extremely satisfied (38.1%, 170/446) with the technology used in group sessions. Most participants were either satisfied or extremely satisfied with the coach facilitators (92.8%, 415/447) and the general group dynamics (78.7%, 350/445). Among those components, fewer than 5% of scores were rated below 3 on the Likert scale. A majority of participants indicated that they were satisfied or extremely satisfied with self-monitoring activities (78.7%, 352/447), the video or DVD resources (88.5%, 386/436), handouts (85.4%, 345/404), and the “virtual package” as a whole (77.9%, 346/444). Further, among these components, fewer than 5% of ratings fell below 3 on the Likert scale.

**Table 5:** Participant satisfaction results^a^.

		Satisfied with the session			Satisfied with home activities and materials				
		Technology (n=446)	Coach (n=447)	Group dynamics (n=445)		Self-monitoring activities (n=447)	Video or DVD (n=436)	Handouts (n=404)	Virtual package (n=444)
Mean (SD)		4.1 (0.9)	4.4 (0.7)	4.1 (0.8)		4.1 (0.9)	4.2 (0.7)	4.2 (0.7)	4.1 (0.9)
**Rating (Likert scale), %**									
	Not at all satisfied 1	2.0	0.2	0.7		1.1	0.7	0.2	0.5
	2	4.5	0.4	4.3		4.9	1.8	2.5	6.1
	3	13.0	6.5	16.4		15.2	8.9	11.9	15.5
	4	42.4	41.0	45.8		38.5	49.8	51	43.7
	Extremely satisfied 5	38.1	52.0	32.8		40.3	38.8	34.4	34.2

^a^Participant satisfaction was rated on a voluntary basis by participants after each virtual group or in-person session.

### Adverse Events

Two hospitalizations occurred during the 6-month trial and both of them were determined by the study physician to be not related to the study. There were no deaths.

## Discussion

### Principal Findings

The purpose of this pilot study was to examine the potential clinical benefit, feasibility, and acceptability of a novel, evidence- and theory-based, technology-assisted behavioral lifestyle intervention for improving health-related quality of life and reducing cardiometabolic risk in ambulatory care. Our main findings suggest that although the eCMP intervention failed to improve health-related quality of life, it showed potential for decreasing cardiometabolic risk among high-risk individuals. Further, the tools and technology-assisted approaches utilized in the intervention demonstrated good feasibility and acceptability among participants.

Comprehensive lifestyle intervention has become a crucial approach to prevention and treatment of obesity [2], metabolic syndrome [32], diabetes [33,34], and cardiovascular disease [35,36]. Previous paradigms for comprehensive lifestyle intervention, which often involve at least weekly in-person one-on-one or group meetings over months to a year, are insufficient to meet the growing population health management needs of the nation. Rising prevalence and suboptimal management of cardiometabolic conditions present a major challenge to the US health care system.

These needs have risen to the national health care agenda, as the Center for Medicare and Medicaid Innovation, through legislation provided within the Affordable Care Act, has specifically called for the use of technology to improve the capacity to provide health services for patients with chronic conditions [37]. The Task Force on Community Preventive Services recommends technology-assisted, multicomponent weight-loss interventions [38]. Several recent studies have demonstrated the potential effectiveness of using digital health tools to promote behavior change and reduce cardiometabolic risk in adults [7,39-50]. Technology may provide the means by which efficacious lifestyle interventions can be translated into real-world, clinic-based settings while retaining effectiveness and increasing access and affordability. In their review, Khaylis et al identified five key components to efficacious technology-based weight loss interventions: use of a structured program, self-monitoring, feedback and communication, social support, and individual tailoring [51]. The eCMP program incorporates all of these elements. The eCMP intervention additionally incorporates several novel elements including semiremote intervention delivery, the use of virtual small groups, and the emphasis on stress management as a distinct but complementary component to diet, physical activity, and behavioral strategies.

Satisfaction with eCMP and the technology-assisted tools was overall high, suggesting good acceptability among participants. The eCMP intervention utilized virtual groups as the primary delivery modality for coach-led face-to-face interactions and curriculum delivery for the stress management and behavioral lifestyle components of the intervention. Technology-assisted interventions for weight loss that incorporate remote intervention delivery and support have been shown to produce outcomes comparable to an in-person intervention [3,40,52,53]. Attendance was generally higher in components utilizing virtual groups compared with the physical activity component that required in-person group visits. This in part may have been due to the timing of the sessions, where virtual groups were offered on weeknights and physical activity in-person sessions were offered on weekends. Technology-assisted approaches to promote behavior change, such as virtual groups, have the potential to improve adherence by making lifestyle interventions more convenient and aiding individuals in overcoming some of the barriers they may encounter in attending frequent, clinic-based, in-person sessions [53,54].

Retention rate was high in our study, compared to intervention randomized controlled trials in primary care settings [55-58]. Overall eCMP attendance was also high, relative to other studies and interventions [59,60] and especially considering the intensity of the intervention. For the 6-month eCMP intervention, 50% attendance correlates to attendance at approximately 15 sessions in 6 months. The novel use of virtual group visits was recently shown to be effective in the delivery of weight management interventions [53,54]. Virtual group visits are a promising approach to increase accessibility of lifestyle interventions to interested individuals [54]. Participants expressed high levels of satisfaction with the technology used in group sessions, self-monitoring activities, and the “virtual package” as a whole. Participants rated group dynamics very highly, suggesting that virtual group format did not hinder the experience of being in an in-person group setting. These findings suggest that it is possible to use technology to increase the scale of an intervention without losing socially important aspects of group-based behavioral lifestyle modification that have been a crucial part of more traditional face-to-face, in-person programs. Further, the participants in this study were older, with varying levels of comfort and skill with technology use. While most participants found the training useful and were able to participate without major difficulties, it may be useful in future interventions to tailor technology training using a pre-assessment of group participants according to baseline skill level, experience, and comfort.

Measurement of health-related quality of life remained unchanged, close to the average score in the general US population [22]. Measures of clinical effectiveness and benefit were overall modest. While weight loss was modest (≤3%) among participants, reductions in waist circumference were significant in the immediate group at 6 months. Also, a majority of participants were enrolled for secondary prevention and were being medically managed for diabetes and/or cardiovascular disease (eg, hypertension and/or dyslipidemia). At baseline, blood pressure and lipid levels were well managed and near goal, making clinical effects of the intervention difficult to discern.

### Limitations

Our study has a number of potential limitations. First, logistical challenges resulted in a change to the study design and primary endpoint. While this change limited our ability to analyze differences between groups after completing the intervention, we were able to assess patterns in trajectory between the two groups at similar timepoints during the course of treatment. Other limitations included a possibility of selective response to the feedback surveys among participants. Participants who responded might have been those who were more committed and more positive toward the intervention. To mitigate this possibility, all participants were highly encouraged by their coaches to complete the survey after each session and all participant evaluations were anonymous. Methodological limitations (eg, small sample size and short follow-up duration) are reflective of a pilot study. Despite these limitations, this study shows that the eCMP intervention is feasible and acceptable in a health care setting and has potential for decreasing cardiometabolic risk among high-risk patients.

### Conclusion

The eCMP intervention showed potential for decreasing cardiometabolic risk among high-risk individuals and also emphasized stress management as a key component. The tools and technology-assisted approaches utilized in the intervention demonstrated good feasibility and acceptability among participants. Future interventions should continue to explore the use of technology to facilitate remote delivery of ambulatory care‒based interventions in order to optimize the partnership of patients and their health care providers in improving lifestyle behaviors.

## Abbreviations

DASH: Dietary Approaches to Stop Hypertension
eCMP: Electronic CardioMetabolic Program
GAD-7: Generalized Anxiety Disorder Scale
NDSR: Nutrition Data System for Research
PHQ-9: Patient Health Questionnaire
